# Notes and News

**Published:** 1891-06-13

**Authors:** 


### NOTES AND NEWS.

COLLECTED AND COMMUNICATED.

The Secretary of State for War has decided that officers of the Army Medical Department shall have the following military titles in future: Surgeon-major-general, surgeon-colonel, brigade-surgeon-lieutenant-colonel, surgeon-lieutenant-colonel, surgeon-major, surgeon-captain, and surgeon-lieutenant. These titles will not, of course, entitle the holders to exercise military command, but will give them all other advantages such as are now enjoyed by military men holding the rank indicated by the second portion of the title. Medical officers will be granted sick leave on the same conditions as those which apply to combatant officers.

The twenty-fourth annual meeting of the Deaconesses' Institution and Hospital, Tottenham, was held last Saturday, Mr. Samuel Figgis presiding. The report stated that fresh work had been undertaken at Woodford, Barrow-in-Furness, Killian, N.B., Rochdale, and Pimlico. During the past year they had admitted 17 Sisters, and there are now a total number of 70 Sisters, of whom 39 are Deaconnesses. The number of patients who last year benefited by their ministrations was 13,587. Expenses have grown larger, but receipts have not materially improved, no legacy or special gift having been received during 1890. The Chairman said that he had inspected the hospital, and had seen the excellent provision made there for the reception of patients of every age and class. Among the wards there was one for paying patients, and he regretted that it was not better known. The report was adopted, and several speakers afterwards bore testimony to the excellent work done by the institution, and to its pressing need for further financial support.

The exertions of the supporters of the Morley House Seaside Convalescent Home for Working Men, at St. Margaret's, Dover, to raise sufficient funds to add and endow fifty beds to the institution, took a public form on Saturday last, when there was a great meeting on behalf of the movement at the Guildhall. The accommodation at the Home has for a long time been inadequate to meet the demands made upon it, and it is now proposed to increase the capacity of the Home by the addition of a new wing, which will, it is estimated, involve an expenditure of £3,500. Towards this sum working men, by special efforts, raised £1,000 during last year, besides clearing off a debt of £600 which embarrassed the Committee. The Lord Mayor, who presided at the meeting, recalled, in his opening address, the circumstances under which the Home was founded in 1883 by the exertions of working-men, aided by Mr. Samuel Morley. Since the Home was first founded the number of those received in it had risen from 100 patients in the first year to 583 during last year. The Earl of Aberdeen proposed a resolution by which the meeting recognised the pressing need for an extension of the accommodation of the Morley Home, and appealed most earnestly to the wealthier classes to assist in raising the sum of £4,000. The Lord Bishop of London seconded the resolution, which was carried. The meeting then terminated.

The annual dinner of the Royal Hospital for Incurables took place last Friday at the Albion Tavern, Aldersgate-street, Sir Whittaker Ellis, M.P., who was accompanied by Lady Ellis, presiding. This charity receives no assistance from the Hospital Sunday Fund, notwithstanding the supplemental work done by the institution by receiving the patients of other hospitals. Four years ago a branch was established at St. Leonards, which has in every way been a successful experiment. In proposing the toast of the evening, the Chairman, alluding to a visit he paid to the hospital, said that he had never seen extreme suffering so admirably relieved. Everything appeared so well organised that he felt that that was the best regulated and best managed of all the charitable institutions he had ever seen. The financial result of the dinner was a contribution of £3,152 to the funds of the institution.

The annual Court of the Governors of the Devon and Exeter Hospital was held on May 28th, under the presidency of Sir John Shelley. The committee's yearly report showed that the number of in-patients treated during 1890 was 1,435, the average cost of each patient being £7 12s. 10d., as compared with £7 2s. 8d. in the previous year. The number of out-patients treated was 2,508. The income for the year was £7,160 4s. 7d., there having been an increase of £35 9s. 6d. in the annual subscriptions, but a falling off in the congregational collections. The total ordinary expenditure was £9,708 5s. 8d., being the largest on record, and there is an actual deficiency on the year's working of £2,548 1s. 1d.; to meet which the committee found it necessary to realise £2,199 4s. stock. It is of the utmost importance that the ordinary income should be raised by at least £1,000 a-year, as there has been for the last ten years a yearly average decrease of capital to the extent of £739. The nurses' home has been completed at a cost of £2,725 1s. 4d., and is now in full occupation, and promises to be both a public boon and a financial success. The Chairman, in moving the adoption of the report, said he hoped the present subscribers would increase their subscriptions. Mr. Andrew seconded, and the report was adopted.

The Marquis of Carmarthen, M.P., who presided at the annual general meeting of the Hospitals Association, held at the Westminster Hospital on Tuesday, in moving the adoption of the seventh annual report, said that the good the institution was capable of doing was incalculable. Its usefulness had been shown during the past year in its preparation of a Bill to amend the Mortmain and Charitable Uses Act, 1888, which, with amicable discussion outside the House of Commons with anyone who might show signs of opposition, would, he had every hope, be got through next year to the great benefit of charities all over the country. He concluded by hoping that the good work which the Association had done in the past might be continued in the future. Dr. Gilbert Smith, in seconding, defined the work of the Association as being twofold: first and chiefest, an educational work, by means of its monthly meetings; and, secondly, a public work, the Association looking in a business way upon the various Bills relating to hospitals, and upon the various public actions in connection with hospitals. The report was adopted. Dr. Steele said that the two most practical outcomes of the Association were the Royal National Pension Fund and the Ambulance Branch. This latter, according to the second annual report presented by Mr. Thomas Ryan, Hon. Secretary of the Branch, has now 46 stations as compared with 39 reported last year. Twelve more out of some 27 suggested by the police, at the request of the Committee, have been approved of, and as soon as the ambulances have been manufactured these will be added to those already in existence, making the total number established by the Hospitals Association 58. A resolution thanking Dr. Bristowe, the President, for his invaluable services in the past was unanimously carried.

